# The experience of Petit Pierre's merry-go-round

**DOI:** 10.1017/S2045796022000750

**Published:** 2023-01-12

**Authors:** Anne Boissière

**Affiliations:** Université Lille Nord de France, Arts, Villeneuve d'Ascq, Hauts-de-France, France

Petit Pierre's merry-go-round is sheltered, maintained and displayed today in the ‘Fabuloserie’ at Dicy in France. This place, which is dedicated to ‘hors norme’ creation, was set up by Alain Bourbonnais (1925–1988), himself an architect, artist and collector: he met Jean Dubuffet with whom he held a correspondence recently published (Dubuffet and Bourbonnais, 2016). But the merry-go-round has also been the subject of a short film, made by Emmanuel Clot (1951–1983) in 1980, that contributed to its reputation at a period where the question of its rescue was asked – this prize-winning film, about seven minutes long, is fortunately available on the internet (Clot). Pierre Avézard called Petit Pierre (1909–1992), was then still alive but ill and placed in a home for the aged. The asset of this film is that one can see the merry-go-round on its original site, at Faye-aux-Loges, near the farmhouse where Petit Pierre lived and worked; moreover, one can see it run by its creator. This dimension of movement or animation is crucial. The merry-go-round – this was the name chosen by Petit Pierre – is not a mere object to be looked at or even to be examined. It moves, delights spectators and makes them dive into childhood again as soon as it runs. Through the movement of his merry-go-round, Petit Pierre *communicates* with people around. We will highlight this aspect of the creation above all.

**Fig. 1.**
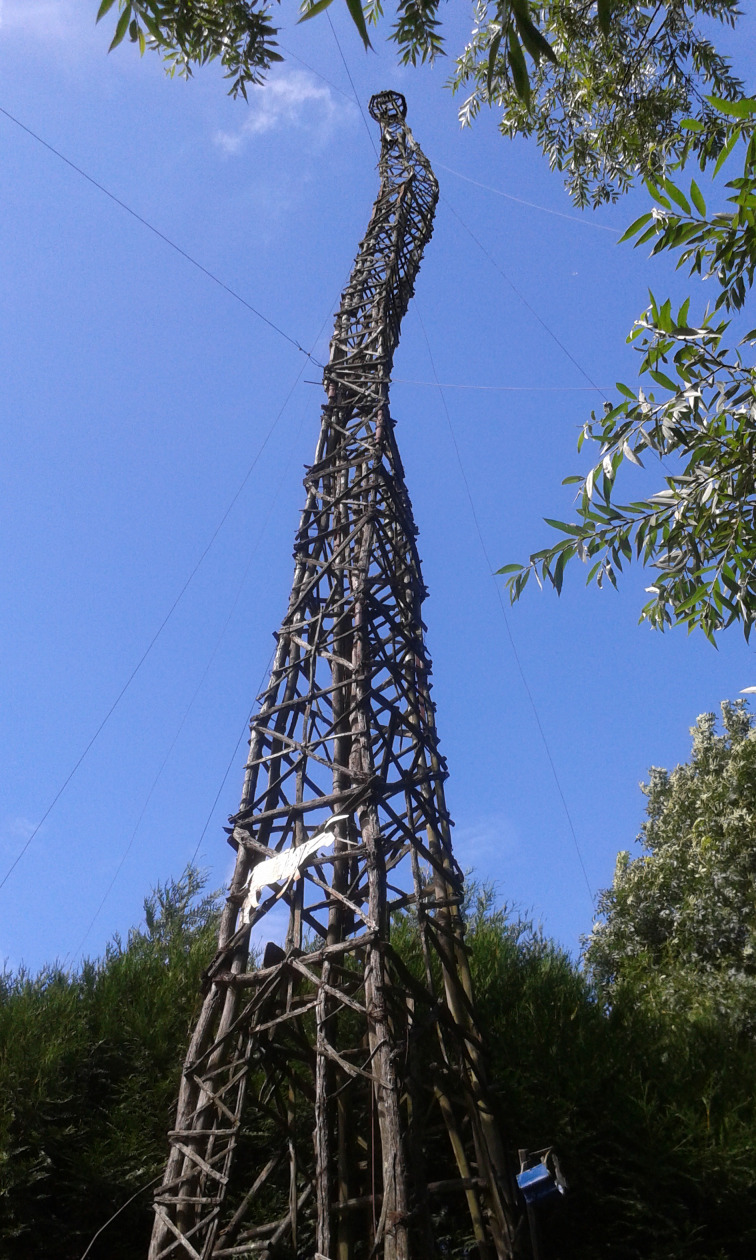
Eiffel Tower, photograph by the author.

Born with a congenital disability – the Treacher-Collins syndrome – which had completely distorted his face, Petit Pierre was practically deaf mute, and he went to school only for two years. His story is linked to the farm in the Loiret where he was placed and where he worked as a cow-herd. However, he belonged to a kindly family and his younger brother Leon, an engineer in aeronautics, took him on journeys to different towns in France and Europe: the merry-go-round evokes them with its planes, its underground train and above all its tremendous Eiffel Tower (Fig. 1), alongside the cows which belonged to Petit Pierre's living environment. Settled in a building of the farmhouse, he began to create his first carousel only with salvaged materials; at the beginning this latter was run with a simple pedalling movement. Progressively, the merry-go-round grew up and got amazing proportions: in its definitive state, on the ground it measures 18 m long, 9.5 m wide and 23 m high, Eiffel Tower included. A little shed, equipped with a motor, was the location where Petit Pierre ran his merry-go-round. Thus every Sunday afternoon, except in winter, during about thirty years, he met people, adults and children, who came visiting him to share this moment of enjoyment, feast and fairy. He set up a car park for visitors, and even when he was in a home for the aged at the end of his life, he went on running his merry-go-round, for which he had a special permission. But the merry-go-round, very fragile, began to decay and it would have completely disappeared without the effort of a whole team, including Petit Pierre's brother Leon, Alain and Caroline Bourbonnais, who accepted to take care of it. The merry-go-round was dismantled and built again, exactly the same, at Dicy: this rescue demanded a very meticulous work. The merry-go-round remains delicate and breakable today. Recently the Eiffel Tower needed to be repaired, which cost a lot: a fundraising, thrown to get money, was a big success which allowed to renew this very unusual construction.

The merry-go-round is an outstanding piece of creation, indeed ‘hors norme’, if it is considered in respect to its production: the complexity of all of the cogs defies the rational mind. It is a do-it-yourself work, without a former plan, and only made with lost materials as sheet metal, rolls and screws. Yet every part, even the smallest, is intertwined with the others. Furthermore, the whole is like a living organism which always demands to be patched up because the lack of one piece entails the whole. It is incredible to see a self-taught man, without particular technical competence, being the author of such an achievement. Nonetheless it is not sufficient to appreciate it in respect to the technical making. The most extraordinary aspect, in fact, comes from the poetry which radiates through this kinetic work, whose status hesitates between architecture and sculpture. Petit Pierre creates a world, *his*, with his cars, his underground and his places, his cows too. All these figures are remarkable for their aliveness, and their author animates them with humour: for instance a figure representing a man unexpectedly sprinkles the spectators with water. The running of the whole is a source of great emotion. It is very hard to keep a distance: one is immediately moved by the merry-go-round when it runs. Through this sensitive dimension of experience, Petit-Pierre, who nearly did not speak, communicated with those who were coming to visit him. Thus the merry-go-round is neither a pure object nor a single technical production. It creates a living and sensitive spatiality as if it were the extension of Petit Pierre's own body. To name this kind of communication through movement and without verbal language, one can introduce the term ‘pathic’ used by the German-born phenomenologist and psychologist Erwin Straus (1891–1975). This means that the sensitive movement, here, is a ‘being-in-the-world’ (Boissière, 2018, 2021). Movement is not synonymous to displacement from one point to another. In this case, it expresses a situation in the world, as it has the status of a living word, although there are no words to be heard. This word is Petit Pierre's own word, which is embodied in the movement of the merry-go-round itself. The French philosopher Henri Maldiney (1912–2013) pointed out this essential dimension of creation, where the author cannot be separated from the deed of creation. Art in the highest meaning of the term is thereby synonymous of ‘existence’: without creation one cannot exist (Maldiney, 2003).

When running, Petit Pierre's merry-go-round is comparable to a dance which takes everybody along through its movement, itself carried by music (*Danser brut*, 2018). Emmanuel Clot's film quite rightly gives a significant place to music which is popular, festive and rhythmic. Petit Pierre also played music with records, when he ran his merry-go-round. Moreover he had a sort of zither on which he loved to play a few notes, as we can see at the end of the film. Rhythm characterises movement: the merry-go-round unfolds itself with its proper time, which is rather measured, unlike current merry-go-rounds that resemble racing cars. Petit Pierre renders poetic his handmade technique. Sense of animation, in the presence of a living rhythm, overcomes the mechanical aspect. Under his hands, do-it-yourself transforms into poetry. The experience of the merry-go-round is not only optical: it is musical and involves the entire body through movement.

**Fig. 2.**
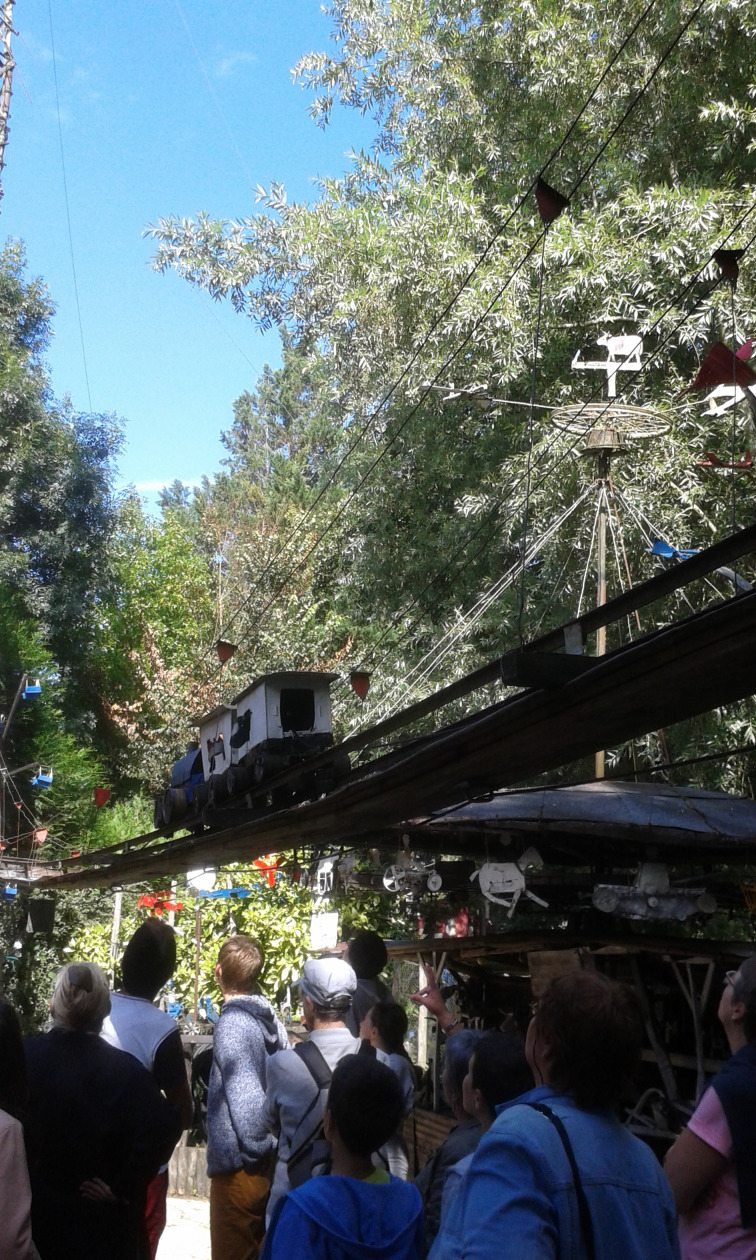
Aerial subway, photograph by the author.

But in fact it is difficult to have the experience of this moving spatiality only through film and screen. Going to the location, in *presence* of the merry-go-round, allows one to point out certain characteristics more precisely. The first one relates to circularity. The merry-go-round, as one imagines it, at least before it was transformed by the technical civilisation, runs round; the movement organises itself around an active centre. Nevertheless the experience of Petit Pierre's merry-go-round does give another impression. As soon as one enters it – of course it is impossible to get on it (Fig. 2), one immediately feels that this is not circular: on one hand its shape is oval, on the other hand the Eiffel Tower at its extreme contributes to disrupt the balance of a circle. This goes round, but at the same time it does not. From the point of view of experience, the organisation of the movement reveals a logic other than the circular one. In fact a poly-rhythm animates the whole. The straight movement is present as well as is the circular one. Moreover, movements come from everywhere: above, behind, alongside, and with all sorts of dimensions. When one hears the merry-go-round starting, it springs from all sides, and this impression of surging is prior, probably stronger and more persistent than circular impressions. The Eiffel Tower soaring up into the air emphasises this impression of a space arising. The poly-rhythm of movement seems to crush the space like a firework. One gets the feeling that the whole might explode. At the same time, one experiences that everything holds fast together, being well attached to the entire system. The merry-go-round appears to be stabilised between two opposite powers, one going inwards and the other outwards. Everything might untie at any moment, but indeed everything persists in all its great fragility and precariousness. The movement verges on the miraculous in carrying on: one can dream and let it go, as children can do when they feel security. One must emphasise that the spatiality is not neutral at all. These movements, having manifold directions, contribute to *animating* the space, similar to what Ludwig Binswanger called ‘directions of meaning’ (*Bedeutungsrichtungen*): through those sensitive dynamisms, a meaning is immediately expressed independently of  verbal language. The spatiality in movement, both sensitive and emotional, is an entire mode of relationship; thereby Petit Pierre's merry-go-round is a mode of communication.

**Fig. 3.**
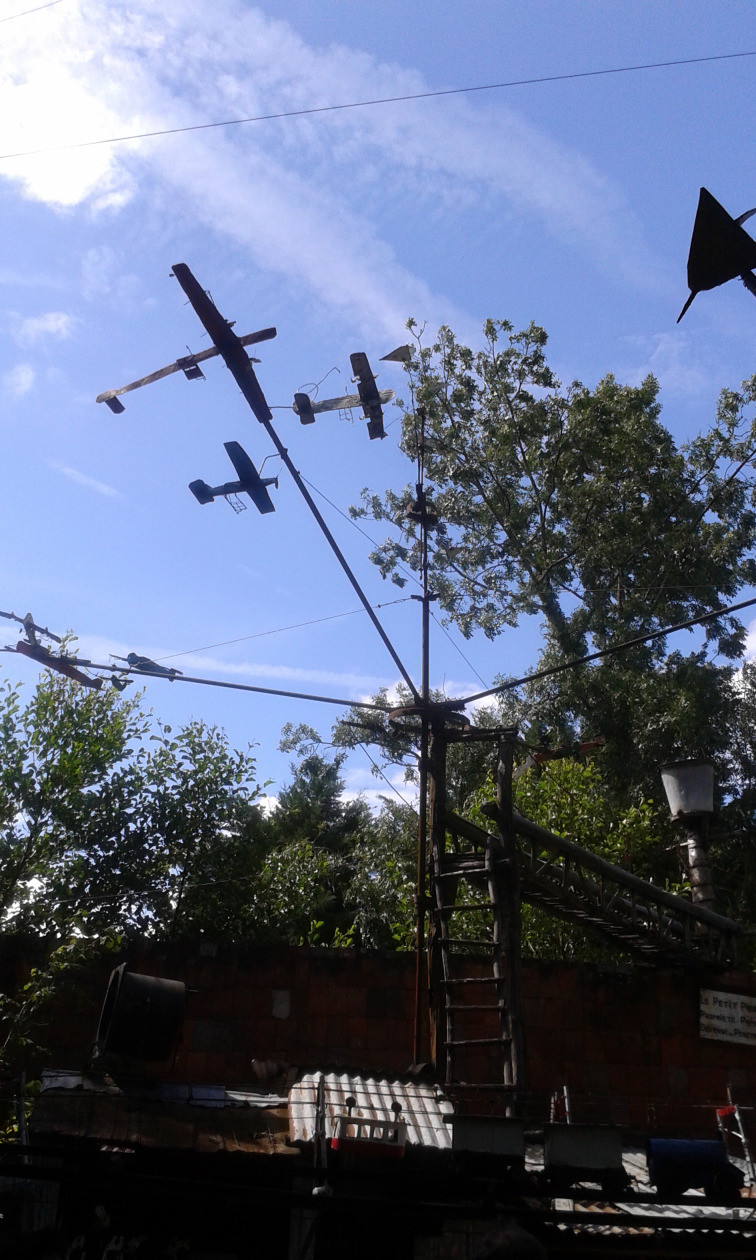
The planes, photograph by the author.

Finally, another characteristic is the aerial. As a sign at the entrance, written by Petit Pierre himself, indicates, the merry-go-round is his home. But this home is open to the sky. It looks as if it were suspended between earth and sky. Speaking about the relationship between Petit Pierre and his carousel, Laurent Danchin introduces the comparison to a spider with its web (Danchin, 2007). The merry-go-round is the equivalent of an architecture made with threads, and it gives the impression to be hung at mid-height, although it is based on the ground. With a precarious balance, its medium is the void; and this gives one a bit of dizziness. Above all it stands out against the vastness of the sky. The gaze rises to see the aeroplanes, but this turns out to be difficult because of the luminosity of the sky (Fig. 3). The merry-go-round has an atmospheric life: if the weather is fine, it is delightful; but if the weather is overcast, it can become very sad. The life of the merry-go-round has something climatic; it changes with the atmosphere of the sky. This permeability with the atmospheric dimension can render it disturbing, similar to the feeling called by Freud ‘uncanny’ (*das Unheimliche*). Of course, the merry-go-round is devoted to enjoyment, but its mode of communication also reveals something which is frightening, through this space which seems too open and susceptible of overwhelming the observer. It contains an excessiveness, perhaps a proximity to death, which is not devoid of sorrow.
